# Resistance-Guided Mining of Bacterial Genotoxins Defines a Family of DNA Glycosylases

**DOI:** 10.1128/mbio.03297-21

**Published:** 2022-03-21

**Authors:** Noah P. Bradley, Katherine L. Wahl, Jacob L. Steenwyk, Antonis Rokas, Brandt F. Eichman

**Affiliations:** a Department of Biological Sciences, Vanderbilt University, Nashville, Tennessee, USA; b Department of Biomedical Informatics, Vanderbilt University School of Medicine, Nashville, Tennessee, USA; c Department of Biochemistry, Vanderbilt University School of Medicine, Nashville, Tennessee, USA; University of Michigan-Ann Arbor; Indiana University Bloomington

**Keywords:** AlkZ, DNA glycosylase, DNA repair, *Streptomyces*, biosynthetic gene cluster, genotoxin, natural product, phylogenetic tree, secondary metabolism, self-resistance, intecalator, DNA cross-link, HTH_42

## Abstract

Unique DNA repair enzymes that provide self-resistance against therapeutically important, genotoxic natural products have been discovered in bacterial biosynthetic gene clusters (BGCs). Among these, the DNA glycosylase AlkZ is essential for azinomycin B production and belongs to the HTH_42 superfamily of uncharacterized proteins. Despite their widespread existence in antibiotic producers and pathogens, the roles of these proteins in production of other natural products are unknown. Here, we determine the evolutionary relationship and genomic distribution of all HTH_42 proteins from *Streptomyces* and use a resistance-based genome mining approach to identify homologs associated with known and uncharacterized BGCs. We find that AlkZ-like (AZL) proteins constitute one distinct HTH_42 subfamily and are highly enriched in BGCs and variable in sequence, suggesting each has evolved to protect against a specific secondary metabolite. As a validation of the approach, we show that the AZL protein, HedH4, associated with biosynthesis of the alkylating agent hedamycin, excises hedamycin-DNA adducts with exquisite specificity and provides resistance to the natural product in cells. We also identify a second, phylogenetically and functionally distinct subfamily whose proteins are never associated with BGCs, are highly conserved with respect to sequence and genomic neighborhood, and repair DNA lesions not associated with a particular natural product. This work delineates two related families of DNA repair enzymes—one specific for complex alkyl-DNA lesions and involved in self-resistance to antimicrobials and the other likely involved in protection against an array of genotoxins—and provides a framework for targeted discovery of new genotoxic compounds with therapeutic potential.

## INTRODUCTION

Bacteria are exceptionally rich sources of secondary metabolites, which are important for their survival and often have therapeutic value. *Streptomyces* produce 35% of all known microbial natural products and nearly 70% of all commercially useful antibiotics, with several being FDA-approved antitumor agents used as first-line cancer treatments (1–4). Secondary metabolites are often toxins used in ecological interactions with other organisms and can target any number of critical cellular functions (5). Natural products that damage DNA (genotoxins) form covalent or noncovalent DNA adducts that can inhibit replication and transcription, undermining genomic integrity through mutagenesis or cell death (6, 7). Consequently, genotoxins are particularly useful antineoplastic agents, as exemplified by several clinically relevant drugs, including doxorubicin, bleomycin, mitomycin C, and duocarmycin analogs (8).

*Streptomyces* produce a wide variety of DNA alkylating and oxidizing agents that have antimicrobial and antitumor properties. Spirocyclopropylcyclohexadienones (duocarmycin A and SA, yatakemycin, and CC-1065) (9, 10), pluramycins (pluramycin A, hedamycin, and altromycin) (11–13), anthracycline glycosides (trioxacarcin A and LL-D49194α1) (14–16), and the leinamycin family (17) contain a single reactive group that covalently modifies purine nucleobases to form a broad spectrum of bulky alkyl-DNA monoadducts. *Streptomyces* also produce bifunctional alkylating agents that react with nucleobases on both DNA strands to create interstrand cross-links (ICLs). Mitomycin C (MMC) from *S. lavendulae* cross-links guanines at their N2 positions, and azinomycin A and B (AZA and AZB) from *S. sahachiroi* and *S. griseofuscus* cross-link purines at their *N*7 nitrogens (18). In addition to alkylating agents, several families of natural products, including bleomycins and enediynes, exert their toxicity by oxidative cleavage of DNA and RNA (19).

The production of secondary metabolites in *Streptomyces* is genetically organized into biosynthetic gene clusters (BGCs), which contain the genes necessary for their biosynthesis, export, regulation, and resistance. Resistance mechanisms protect antibiotic producers from toxicity of their own natural products and include toxin sequestration, efflux, modification, destruction, and target repair/protection (20, 21). In the case of genotoxins, several DNA repair enzymes have been identified as target repair resistance mechanisms, including direct reversal of streptozotocin alkylation by AlkB and AGT (alkylguanine alkyltransferase) homologs (22), base excision of yatakemycin-adenine adducts by the DNA glycosylase YtkR2 (23, 24), nucleotide excision of DNA adducts of several intercalating agents, including daunorubicin (25), and putative replication-coupled repair of distamycin-DNA adducts (26).

The AZB gene cluster in *Streptomyces sahachiroi* encodes a DNA glycosylase, AlkZ, which unhooks AZB-ICLs and provides cellular resistance against AZB toxicity (27, 28). ICL unhooking by AlkZ involves hydrolysis of the N-glycosidic bonds of the cross-linked deoxyguanosine residues, producing abasic (AP) sites that can be repaired by the base excision repair pathway (29). AlkZ belongs to the relatively uncharacterized HTH_42 superfamily of proteins found in antibiotic-producing and pathogenic bacteria (28). The crystal structure of AlkZ revealed a unique C-shaped architecture formed by three tandem winged helix-turn-helix motifs, with two catalytically essential glutamine residues within a QΦQ motif (Φ is an aliphatic residue) located at the center of the concave surface (30). We recently characterized a second HTH_42 protein from Escherichia coli, YcaQ, as a DNA glycosylase that excises several types of *N*7-alkylguanine ICLs and monoadducts using a catalytic QΦD motif and that functions as a secondary pathway to nucleotide excision repair for bacterial resistance to the nitrogen mustard mechlorethamine (31).

The targeted discovery of natural products has been employed to search for novel scaffolds in plants, fungi, and bacteria and can be useful for identifying specific classes of compounds (32–34). Genome mining can be used to search for unidentified BGCs through analysis of core/accessory biosynthetic genes (PKS, NRPS, and tailoring enzymes), comparative/phylogeny-based mining, regulatory genes, and, more recently, resistance genes (35). Some of these resistance-based mining approaches focus on the experimental screening of antibiotic resistance, while others rely on bioinformatic tools to identify resistance genes within clusters based on homology to known resistance genes (36–39). However, many of these resistance-based methods have not been applied in bacteria for targeted discovery.

Here, we characterized the genomic differences of the HTH_42 proteins found in 435 species of *Streptomyces* to develop additional insight into this new family of DNA repair proteins and applied this information in resistance-guided genome mining to characterize unknown BGCs or identify new genotoxins. We found that these proteins fall into two distinct subfamilies that are delineated by amino acid sequence, genomic context, and copy number. Proteins similar to *S. sahachiroi* AlkZ (AlkZ-like, AZL) are highly variable in sequence and enriched in BGCs, many producing known genotoxic alkylating agents. We show that the AZL protein within the BGC of the known DNA alkylating agent hedamycin (HED) is a resistance DNA glycosylase specific for HED-guanine lesions, consistent with AZL-mediated DNA repair activity as a general self-resistance mechanism to genotoxins in antibiotic producers. Moreover, we found AZL proteins in BCGs that are either uncharacterized or that produce natural products not previously known to be genotoxic, validating resistance genome mining as an approach to discover new genotoxins. In contrast, E. coli YcaQ-like (YQL) proteins are highly conserved in sequence and genetic neighborhood and are not associated with BGCs. We show that like E. coli YcaQ, two YQL enzymes from Actinobacteria have weaker substrate specificity than AZL proteins, suggesting a broader role of this subfamily of HTH_42 proteins outside antibiotic self-resistance in bacteria.

## RESULTS

### YQL and AZL proteins in *Streptomyces* are evolutionarily distinct.

**FIG 1:**
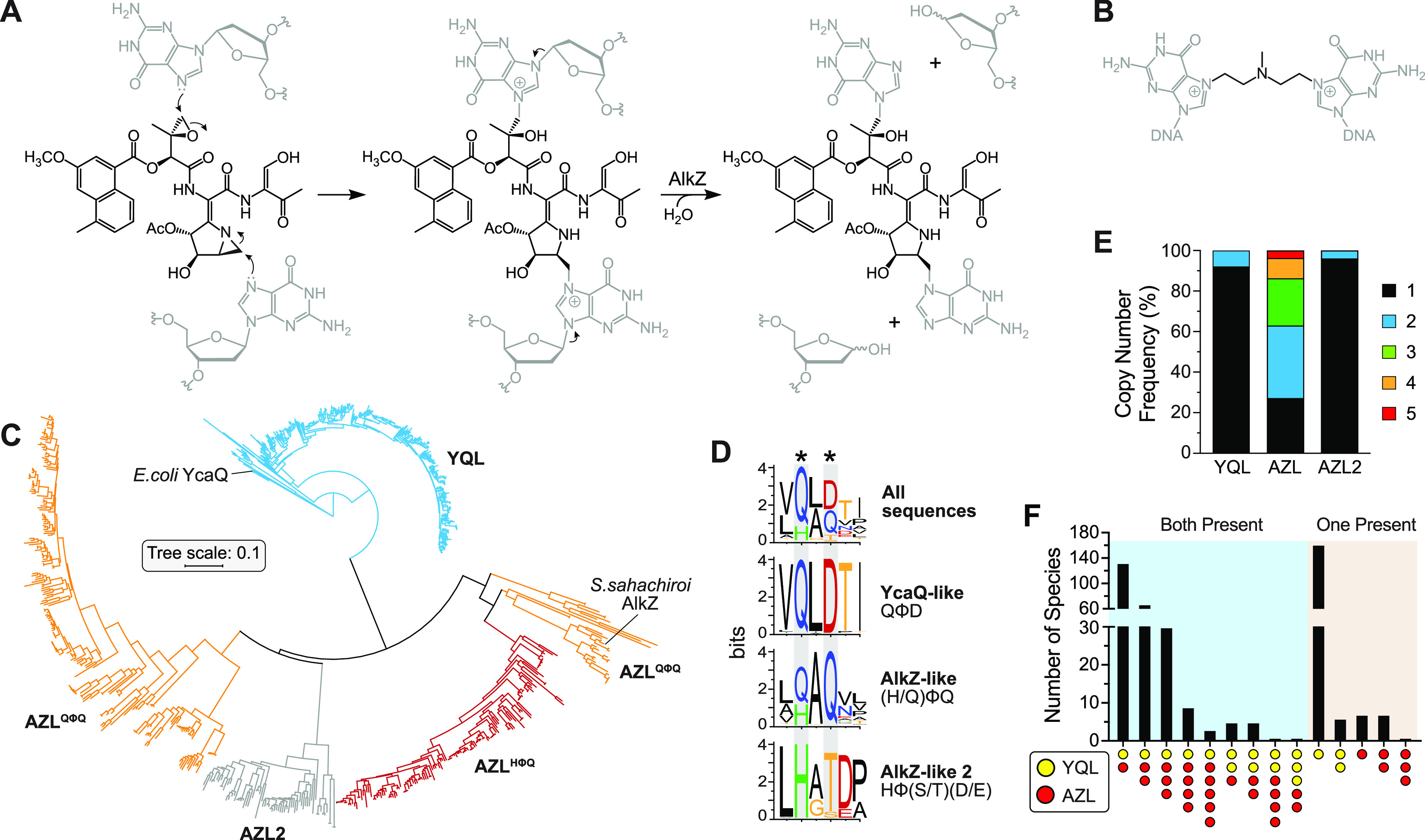
Phylogenetic organization of YQL/AZL proteins in *Streptomyces*. (A) Azinomycin B reacts with opposite strands of DNA to form an ICL, which is unhooked by AlkZ. (B) Structure of a nitrogen mustard ICL derived from mechlorethamine and unhooked by E. coli YcaQ. (C) Phylogenetic tree of YcaQ-like (YQL, blue) and AlkZ-like (AZL, red/orange; AZL2, gray) *Streptomyces* proteins (*n* = 897). The red and orange AZL clades distinguish HΦQ and QΦQ catalytic motifs. E. coli YcaQ and *S. sahachiroi* AlkZ proteins are labeled. (D) Sequence logos for the catalytic motifs in YQL, AZL, and AZL2 proteins. Catalytic residues are marked with asterisks. Colors correspond to side chain chemistry. (E) Copy number frequency per *Streptomyces* genome as a percentage of the total species analyzed (*n* = 436 species, 897 sequences). One-way ANOVA significance (*P*) values of copy number variance are 0.0078 (YQL-AZL), 0.0033 (AZL-AZL2), and 0.3305 (YQL-AZL2), the latter of which is not significant. (F) YQL/AZL coincidence frequency. The blue-shaded section represents species containing both subfamilies; the tan-shaded section represents species containing either YQL or AZL.

E. coli YcaQ and *S. sahachiroi* AlkZ are the only characterized members of the HTH_42 superfamily and are unique in their ability to unhook ICLs and to provide cellular resistance to cross-linking agents. Both enzymes fully unhook ICLs derived from AZB (Fig. 1A). While AlkZ is specific for AZB-ICLs and is essential to the AZB-producing organism, YcaQ unhooks a broader range of ICLs, including those derived from the simple bifunctional alkylating agent mechlorethamine (Fig. 1B), and displays robust excision activity for *N*7-methylguanine (7mG) monoadducts (28, 30, 31). YcaQ and AlkZ belong to one of five classes of HTH_42 proteins characterized by domain organization, which accounts for >95% of all HTH_42 proteins (see Fig. S1A in the supplemental material). Approximately two-thirds of the known HTH_42 proteins in prokaryotes are found in Actinobacteria, with ∼25% of those sequences from *Streptomycetales* (Fig. S1B and C). The remainder are found in several different orders of Bacteria and a very small number (12) in Archaea.

10.1128/mbio.03297-21.1 FIG S1 HTH_42 superfamily taxonomy, phylogeny, and copy number analysis. (A) Domain schematics for top 5 Pfam classes of HTH_42 superfamily proteins. Number of sequences for each organization is labeled to the right, along with the domain key. Dark vertical lines represent predicted unstructured regions. (B) Taxonomic distribution of HTH_42 proteins from prokaryotes (class, 4,797 sequences). (C) Taxonomic distribution of HTH_42 proteins from Actinobacteria (order, 3,033 sequences). (D) Sequence alignment of YcaQ-like (YQL, blue), AlkZ-like (AZL, red; AZL2, yellow) proteins in the region surrounding the catalytic motif (asterisks). E. coli YcaQ and *S. sahachiroi* AlkZ are shown at the top of each block as a reference. (E) Denaturing PAGE of 5′-Cy5 labeled d7mG-DNA substrate (S) and nicked AP-DNA product (P) after treatment with either buffer (mock), E. coli YcaQ, *S. sahachiroi* AlkZ, or Streptomyces caeruleatus AZL2. AP-DNA resulting from glycosylase activity was treated with 0.2 M NaOH to generate β,δ-elimination products, which are quantified below the gel. Download FIG S1, EPS file, 1.1 MB. Copyright © 2022 Bradley et al. 2022 Bradley et al. https://creativecommons.org/licenses/by/4.0/ This content is distributed under the terms of the Creative Commons Attribution 4.0 International license.

To better understand the evolutionary and phylogenetic breadth of this superfamily in *Streptomyces*, we collected and analyzed all HTH_42 protein sequences from available genomes using a combination of BLAST searches against *Streptomyces* genomes in GenBank and HHMR protein domain searches of the BLAST hits against the Pfam database (Table S1). Alignment of the 897 sequences showed that YQL and AZL proteins fall into distinct clades that represent 49% and 43% of the total number of sequences, respectively (Fig. 1C). The clades are defined in part by unique catalytic motifs QΦD (YQL) and (Q/H)ΦQ (AZL), where Φ is an aliphatic residue (30, 31). YQL proteins show a high degree (>75%) of amino acid sequence conservation, whereas the AZL subfamily is more diverse, with only ∼40% amino acid similarity on average. The differences in conservation are consistent with mutation rates as approximated by tip-to-root branch lengths (0.23 for YQL and 0.59 for AZL). In addition, we found that 8% of sequences do not fall into either YQL or AZL clades and contain a unique catalytic consensus sequence, HΦ(S/T)(D/E) (Fig. 1C and D). Because these sequences exhibit greater sequence similarity overall to AZL than YQL, we refer to this third homolog as AZL2. Interestingly, AZL2 is more similar to YQL in its copy number and genomic location (see below) and, thus, is somewhat of a hybrid between AZL and YQL. We verified that proteins within the AZL2 clade contain bona fide DNA glycosylase activity, as the *S. caeruleatus* AZL2 protein excised 7mG from DNA in a manner similar to that of *S. sahachiroi* AlkZ (Fig. S1E).

10.1128/mbio.03297-21.4 TABLE S1 List of HTH_42 proteins by organism. List of all *Streptomyces* YQL (YcaQ-like), AZL (AlkZ-like), or AZL2 (AlkZ-like 2) proteins in this study, along with the GenBank/RefSeq genome/assembly ID for each organism. Homologs are alphabetized by organism. Download Table S1, XLSX file, 0.04 MB. Copyright © 2022 Bradley et al. 2022 Bradley et al. https://creativecommons.org/licenses/by/4.0/ This content is distributed under the terms of the Creative Commons Attribution 4.0 International license.

Another striking difference between the YQL and AZL families is that AZL genes are often found in multiple copies and in different combinations in many species of *Streptomyces*. The copy number differences between the different clades are significant, with the majority (90 to 95%) of YQL and AZL2 homologs found as a single copy and AZL mainly found in multiple (2–5) copies (Fig. 1E). The coincidence of YQL and AZL also varies. Although the most common combination is the presence of a copy of each YQL and AZL, many other combinations are observed (Fig. 1F). The number of species that contain both genes decreases as the copy number increases. For species containing either YQL or AZL (not both), the majority contain a single YQL copy, with just a few species having only AZL present. These results show that both YQL and AZL proteins are broadly distributed across *Streptomyces* and are distinct with respect to sequence, diversity, and copy number.

### AZL proteins are prevalent in biosynthetic gene clusters.

**FIG 2:**
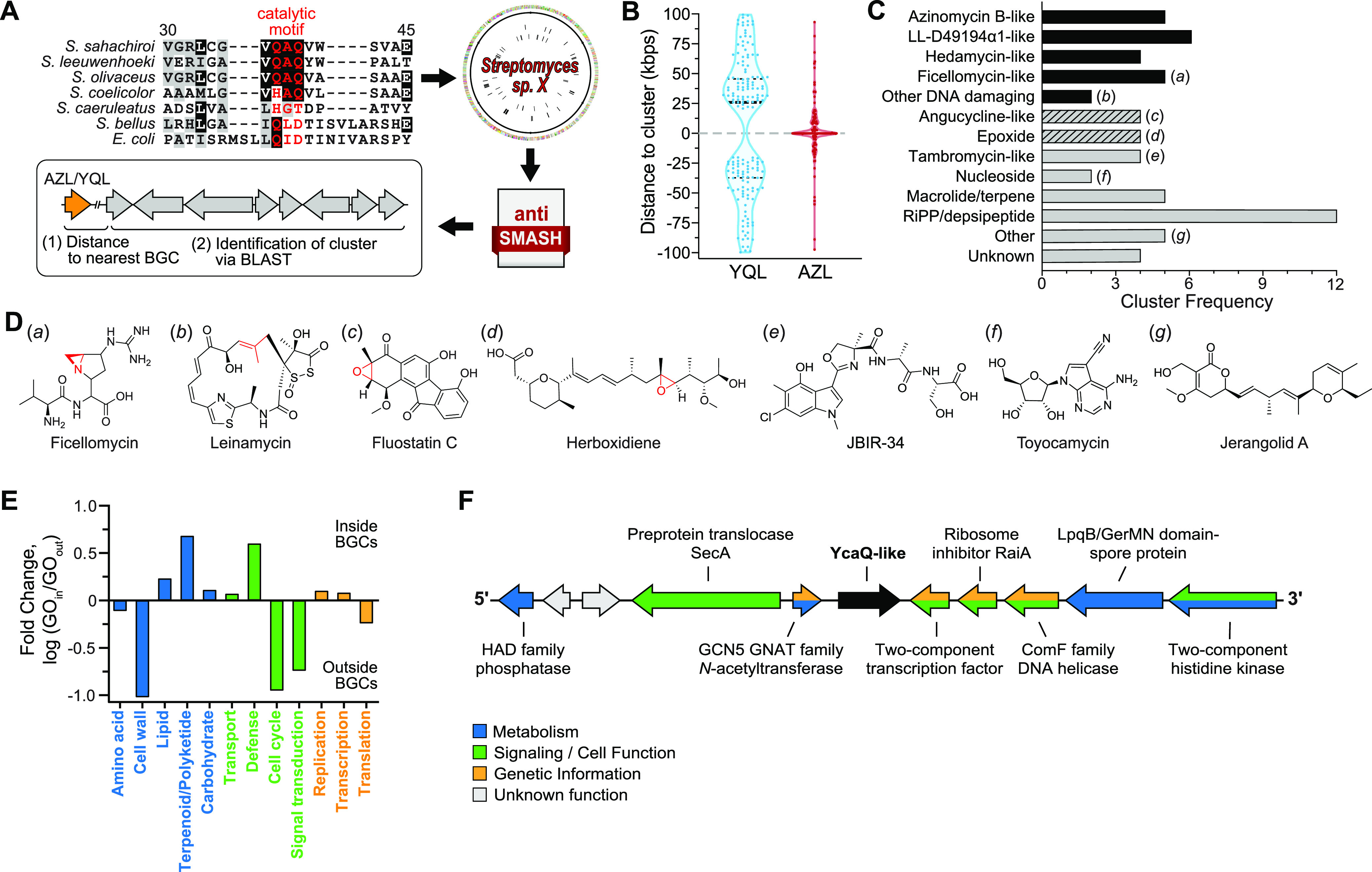
*Streptomyces* AZL proteins are found in diverse uncharacterized biosynthetic gene clusters. (A) Schematic depicting the workflow for identification of HTH_42 homologs in uncharacterized *Streptomyces* BGCs. Homologs were identified through the presence of the catalytic motif (red text in sequence alignment). The amino acid numbering is in relation to *S. sahachiroi* AlkZ. The corresponding *Streptomyces* genomes were input into antiSMASH, from which genomic distances between YQL/AZL and the nearest BGC as well as homologous clusters were extracted. (B) Violin plot showing the distribution of distances of YQL (*n* = 167) and AZL (*n* = 154) genes to the nearest BGC (in kbp; ±100 kb). The dotted line at 0 kb represents the 5′ (+)/3′ (−) termini of the nearest BGC. Thick and thin dashed lines within the plot represent the median and upper/lower quartiles, respectively. The chi-square significance (*P*) value between YQL and AZL data is less than 0.0001. (C) Frequency of various types of BGCs in which AZL genes were found (*n* = 68 clusters identified). The *y* axis denotes the natural product/scaffold type to which that cluster is most homologous. Black bars represent known DNA alkylators or DNA interacting metabolites, and hashed bars represent potential DNA-damaging metabolites. Lowercase letters to the right of the bars correspond to structures shown in panel D. (D) Representative compounds corresponding to BGC types in panel C. Potential reactive sites are colored red. LL-D4919α1 and hedamycin structures are shown in Fig. 3. (E and F) Nearest-neighbor analysis of AZL (E) and YQL (F). (E) Nearest genes to AZL proteins found inside and outside clusters, shown as the ratio of GO terms inside and outside and grouped by function (blue, metabolic; green, cell signaling and function; orange, genome maintenance). (F) Representative example from Streptomyces griseoviridis of nearest neighbor analysis for YQL proteins. Genes are colored according to function as in panel E (gray, unknown/hypothetical gene). These genes are invariant for all YQL proteins, with the exception of the outermost genes, in which only one instance of variance was observed.

Given the distinct phylogeny of YQL and AZL proteins, we next examined their proximity to BGCs and characterized the identities of clusters containing a putative homolog. To perform this analysis, we identified all BGCs in the genomes of known *Streptomyces* species containing an HTH_42 protein, determined the most similar known cluster via BLAST, and extracted the distance in base pairs between the YQL/AZL gene and the nearest 3′ or 5′ end of each BGC (Fig. 2A and Table S2). Strikingly, none of the 442 YQL genes localize to within 20 kb of the most proximal gene cluster in that organism (Fig. 2B). In contrast, AZL genes are primarily found inside or in close genomic proximity to clusters, with an average distance of roughly 2.3 kb from the nearest BGC (compared to 25 kb for YQL). Despite their sequence similarity to AZLs, the AZL2 proteins are more like YQL in that they also are not observed within 20 kb of a BGC (Table S2).

10.1128/mbio.03297-21.5 TABLE S2 Proximity of HTH_42 proteins to biosynthetic gene clusters. Results from BGC proximity analysis organized by AZL and YQL protein distances to the nearest antiSMASH-predicted cluster in the species’ genome. Nearest cluster upstream [5′/(−)] and/or downstream [3′/(+)] is recorded with the cluster ID, and the most related cluster BLAST hit is denoted with the percent gene similarity. No BGC identified denotes the cluster BLAST could not find a similar cluster which compares to the hit by homology. Download Table S2, XLSX file, 0.06 MB. Copyright © 2022 Bradley et al. 2022 Bradley et al. https://creativecommons.org/licenses/by/4.0/ This content is distributed under the terms of the Creative Commons Attribution 4.0 International license.

We found that AZL proteins are particularly enriched in uncharacterized *Streptomyces* BGCs, with 68 homologs localizing within a variety of different types of clusters (Fig. 2C and D and Table S3). Almost half (*n* = 32; 47%) localize to clusters resembling those producing known DNA-damaging agents, including AZB (*n* = 5), LL-D4919α1 (LLD, *n* = 6), HED (*n* = 4), ficellomycin/vazabitide A (*n* = 5), and C-1027/leinamycin (*n* = 2) (12, 16–18, 40, 41). In addition, several other clusters are related to potential DNA-damaging agents on the basis of a reactive epoxide functional group in the natural product, including angucycline-like molecules (*n* = 4) herboxidiene and asukamycin. The remaining 10 uncharacterized BGCs are related to clusters that produce macrolides/terpenes, tambromycin-like compounds, and various RiPPs/depsipeptides (Fig. 2C and D).

10.1128/mbio.03297-21.6 TABLE S3 AZL proteins found in characterized and uncharacterized biosynthetic gene clusters. %I/S to AlkZ (column C) is the percent identity or similarity to *S. sahachiroi* AlkZ. Cluster BLAST (column E) is the most similar BGC as determined by cluster BLAST analysis (% similarity is the percentage of genes in uncharacterized BGC that have homology to genes in the known similar BGC). Download Table S3, XLSX file, 0.01 MB. Copyright © 2022 Bradley et al. 2022 Bradley et al. https://creativecommons.org/licenses/by/4.0/ This content is distributed under the terms of the Creative Commons Attribution 4.0 International license.

Bacterial genes of similar function or in a particular pathway are frequently clustered into neighborhoods or operons within the genome; thus, we investigated the nearest neighbors of *Streptomyces* YQL and AZL genes. We collected gene ontology (GO) terms describing the biological functions of the five nearest neighbors on either side of 40 YQL genes, 40 AZL genes inside BGCs, and 40 AZL genes outside BGCs, which collectively represent ∼15% of the total of all homologs. Biological processes were grouped into three categories: metabolism, signaling/cell function, and genetic information processing. Several key differences were found between the neighborhoods of AZL genes inside versus outside clusters (Fig. 2E and Fig. S2). AZL genes within BGCs were more often found near terpenoid/polyketide/nonribosomal protein synthesis and resistance/defense genes. The defense genes fell into several types: ABC transporters/permeases, α/β-fold hydrolases (VOC resistance proteins), DinB DNA-damage inducible hydrolases, and other AZL proteins. For those AZL genes found outside BGCs, there is an abundance of neighbors involved in cell wall biosynthesis, cell cycle control, and signal transduction. In contrast, there were no significant differences between AZL neighbors involved in processing genetic information inside versus outside clusters (Fig. 2E). In contrast to the variation in the function of AZL gene neighbors, the functions of YQL neighbors (outside clusters) are nearly invariant and are composed of a variety of different gene types with no apparent functional connection between them (Fig. 2F). The functions of many of these neighbors have not been elucidated in *Streptomyces*, but some are homologous to N-acetyltransferase, a two-component transcription factor/histidine kinase, and a DNA helicase (ComF) involved in transformation competence. Thus, both the sequences and the genomic neighborhoods of YQL proteins are relatively conserved and always found outside of BGCs, in contrast to the more variable copy number, sequence, and neighborhood of AZL genes prevalent within BGCs.

10.1128/mbio.03297-21.2 FIG S2 Nearest neighbor analysis of AZL proteins. Gene ontology (GO) analysis for AZL nearest neighbors (±5 open reading frames) inside (A) and outside (B) BGCs. Venn diagrams depict the number of neighbors involved in metabolism (blue), signaling and cell function (green), and processing of genetic information (orange). The boxes represent subdivisions of each of the three functions, colored with respect to the key below. Uncharacterized/hypothetical proteins (40 inside, 90 outside) that could not be identified by homology are not included in these data. Full GO term analysis can be found in Tables S5 to S7. Download FIG S2, EPS file, 0.9 MB. Copyright © 2022 Bradley et al. 2022 Bradley et al. https://creativecommons.org/licenses/by/4.0/ This content is distributed under the terms of the Creative Commons Attribution 4.0 International license.

### Characterized BGCs containing AZL proteins.

**FIG 3:**
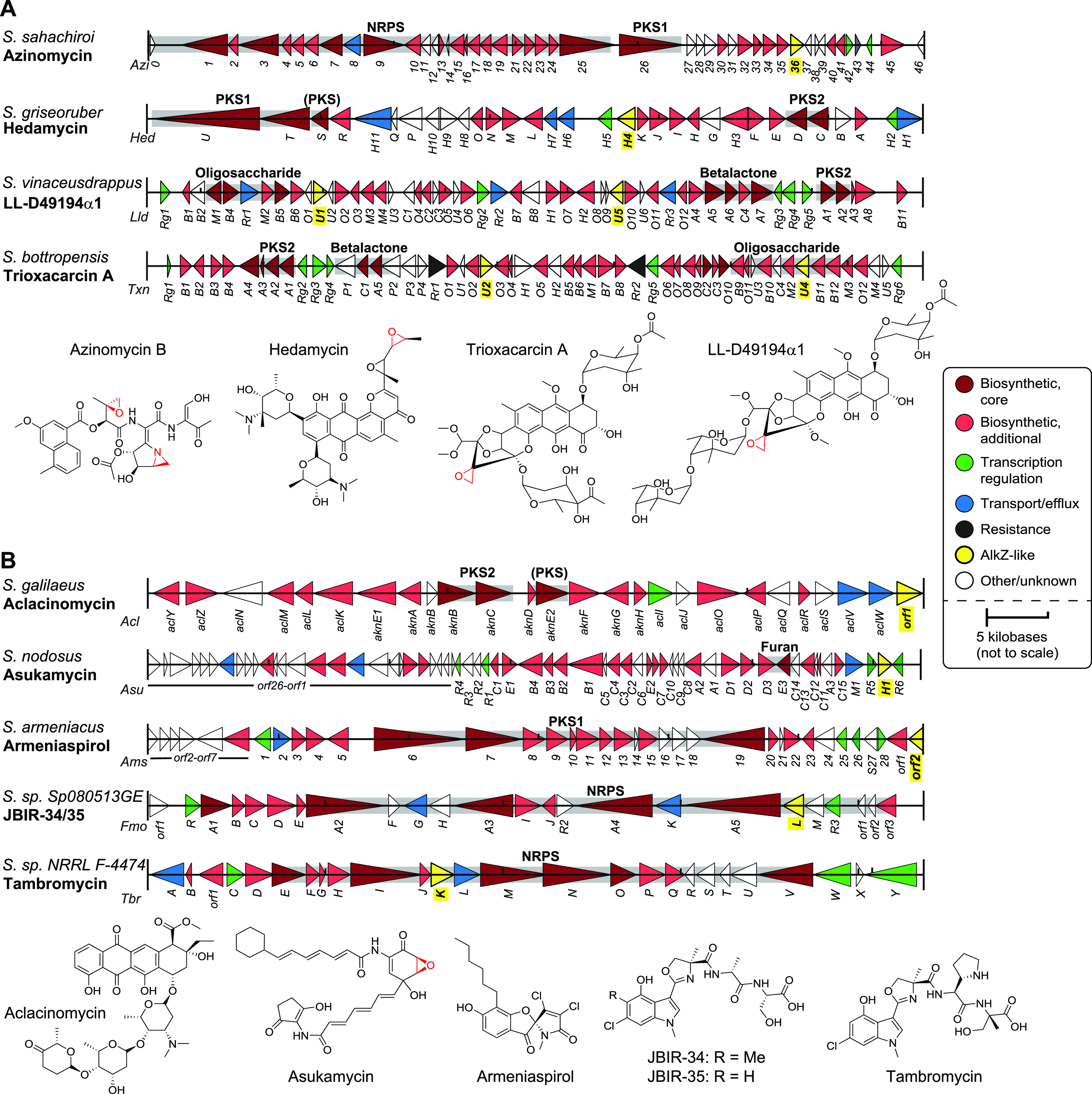
AZL proteins found in characterized *Streptomyces* biosynthetic gene clusters. (A and B) Gene diagrams for AZL-containing BGCs producing DNA alkylating agents (A) and compounds not known to alkylate DNA (B). Gene names are labeled below the cluster diagrams. The biosynthetic scaffold produced by specific genes in the cluster is shaded gray and labeled above the respective genes. NRPS, nonribosomal peptide synthetase; PKS1/PKS2, type 1/2 polyketide synthase; (PKS), PKS-like. Chemical structures of the metabolites produced by each cluster are shown at the bottom of each panel.

With the discovery that a significant proportion of AZL proteins reside within BGCs, we took a closer look at the nine characterized BGCs identified to contain an AlkZ homolog in the MIBiG database (Table S3). Four of these produce known DNA-alkylating agents (Fig. 3A), which contain reactive epoxide moieties like AZB that are scaffolded on diverse natural product backbones (Fig. 3A). Whereas AZB is a bifunctional alkylating agent, HED, trioxacarcin A (TXNA), and LL-D49194α1 (LLD) are monofunctional alkylating agents that react with nitrogen N 7 of guanine in specific nucleotide sequences via their epoxide rings and also intercalate the DNA helix via their planar ring systems (12, 42). TXNA and LLD clusters each contain two AlkZ paralogs (TxnU2/U4 and LldU1/U5), whereas the HED cluster contains one (HedH4) that resides between the two polyketide synthase genes.

The remaining five AZL-containing clusters in MIBiG produce compounds that are not known to alkylate DNA but that share some structural characteristics with the alkylating agents described above (Fig. 3B). Aclacinomycin contains an anthracycline core surrounded by sugars that allow it to intercalate into DNA and act as a topoisomerase I poison, potentially generating downstream DNA damage (43). Asukamycin contains a modified PKS scaffold and an electrophilic epoxide ring and has been shown to act as both a farsenyltransferase inhibitor and a molecular glue between the UBR7 E3 ubiquitin ligase and the TP53 tumor suppressor, leading to cell death (44, 45). Armeniaspirol contains a unique chlorinated pyrrole and inhibits the AAA+ proteases ClpXP and ClpYQ, leading to cell division arrest in Gram-positive bacteria (46). The other two BGCs produce compounds of known structure but unknown function. Tambromycin and JBIR-34/35 are similar NRPS compounds containing densely substituted chlorinated indole and methyloxazoline moieties (47). The presence of AZL proteins in these clusters suggests that these compounds are genotoxins or otherwise react with DNA and/or that these particular AZL homologs have a function outside DNA repair.

### The AZL protein within the HED BGC is a DNA glycosylase specific for HED-DNA lesions and provides cellular resistance to HED toxicity.

**FIG 4:**
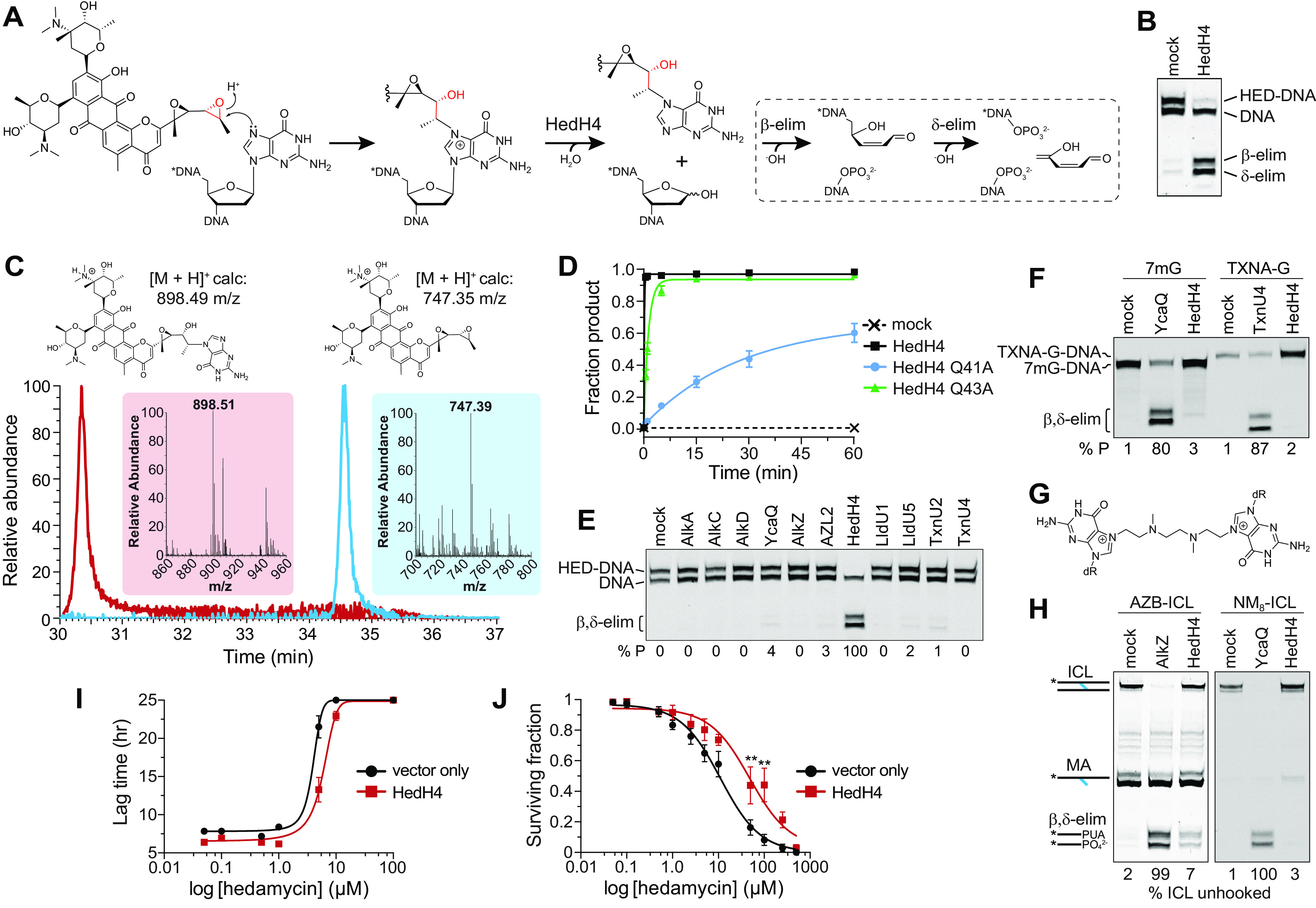
HedH4 excises hedamycin-guanine adducts from DNA and provides cellular resistance to hedamycin toxicity. (A) HED modification of deoxyguanosine in DNA forms a HED-DNA adduct that is hydrolyzed by HedH4 to generate an abasic (AP) site in the DNA and free HED-guanine. The reactions within the dashed line are not catalyzed by HedH4. The AP nucleotide is susceptible to base-catalyzed nicking to form shorter DNA products containing either a 3′-phospho-α,β-unsaturated aldehyde (PUA; β-elimination) or a 3′-phosphate (δ-elimination). The asterisk denotes the original 5′-end of the DNA. (B) Denaturing PAGE of 5′-Cy5-labeled HED-DNA substrate and β- and δ-elimination products after treatment with enzyme or buffer (mock) for 1 h, followed by NaOH to nick the AP site. The HED-DNA reaction only goes to ∼50% completion under our reaction conditions, as shown by the two bands of equal intensity in the mock reaction. (C) HPLC-MS analysis of HED (blue) and the HED-guanine excision product from reaction of HedH4 and HED-DNA (red). Axis represents elution time (*x*–axis) versus relative abundance from total ion count (*y*–axis). Insets show mass spectra of each elution peak. (D) Wild-type and mutant HedH4 glycosylase activity for HED-DNA. Spontaneous depurination from a no-enzyme reaction (mock) is shown as a negative control. Data are means ± standard deviations (SD) (*n* = 3). Curves were fit to a single exponential. Representative data are shown in Fig. S3C. (E) Denaturing PAGE of HED-DNA adducts after 1 h of incubation with either buffer (mock) or bacterial alkylpurine-DNA glycosylases. (F) Denaturing PAGE of 1-h reaction products of E. coli YcaQ and HedH4 with 7mG-DNA (left) and *S. bottropensis* TxnU4 and HedH4 with TXNA-DNA (right). (G) Structure of NM_8_-ICL. (H) Denaturing PAGE of AZB-ICL unhooking by *S. sahachiroi* AlkZ and HedH4 (left) and NM_8_-ICL unhooking by E. coli YcaQ and HedH4 (right). Reactions were treated with buffer (mock) or enzyme for 1 h, followed by alkaline hydrolysis. MA, monoadduct. (I) HED inhibition of E. coli K-12 transformed with *hedH4*/pSF-OXB1 (constitutively expressed) or empty vector pSF-OXB1. The lag time is defined as the time elapsed before cells start to grow exponentially. Data are means ± SD (*n* = 3). Growth curves are shown in Fig. S3F and G. Significance values were determined by unpaired *t* test of the mean lag time values (*, 0.05 ≤ *P* ≤ 0.01; ***, 0.001 ≤ *P* ≤ 0.0001). (J) Colony dilution assay for E. coli strains with or without HedH4 exposed to increasing concentrations of HED for 1 h. Surviving fraction (%) is relative to untreated cells. Values are means ± SD (*n* = 3). Significance values were determined by unpaired *t* test of the mean sensitivity values (*, 0.05 ≤ *P* ≤ 0.01; **, 0.01 ≤ *P* ≤ 0.001).

The *alkZ* gene embedded within the AZB BGC provides exquisite resistance to the potent cytotoxicity of this natural product (27, 28). To determine if AlkZ homologs other than those in the AZB BGC provide self-resistance to their cognate natural products, we characterized the DNA glycosylase and cellular resistance activities of HedH4 for HED-DNA adducts. HED is a potent antibiotic/antitumor agent that induces a strong DNA damage response (48). The bisepoxide side chain alkylates the *N*7 position of guanines in 5′-(C/T)G sequences (Fig. 4A), the highly oxidized aromatic polyketide intercalates the DNA helix, and two C-glycosidic linked aminosugars interact with the minor groove (12). We generated site-specifically labeled HED-guanosine adducts in DNA by reacting purified compound with an oligonucleotide containing a HED target sequence, d(TGTA). The HED-DNA adduct was stable relative to other *N*7-alkylguanine lesions as judged by thermal depurination (Fig. S3B) (31, 49). We first assessed the ability of purified HedH4 to hydrolyze HED-DNA using a gel-based glycosylase assay that monitors alkaline cleavage of the AP site product (30, 31). Reaction of HedH4 with HED-DNA followed by hydroxide work-up resulted in β- and δ-elimination products, consistent with production of an AP site from DNA glycosylase-mediated excision of the N-glycosidic bond of the HED-guanosine nucleotide (Fig. 4A and B). We verified the identity of the excision product as HED-guanine by high-performance liquid chromatography-mass spectrometry (HPLC-MS) (Fig. 4C). To verify that the HED-guanine product was not generated by a contaminating enzyme and to examine the conservation of the catalytic QΦQ motif, we purified alanine mutants of the two glutamine residues and tested their activity under single-turnover conditions (Fig. 4D and Fig. S3A and C). The calculated rate constant (*k*_cat_) for wild-type HedH4 was at least 7.8 ± 0.5 min^−1^ (the reaction was complete at the earliest time point). Relative to the wild type, the Q41A mutant was at least 225-fold slower (*k*_cat_ = 0.04 ± 0.01 min^−1^) and the Q43A mutant at least 10-fold slower (*k*_cat_ = 0.8 ± 0.2 min^−1^), indicating that both Gln residues in the HedH4 QΦQ play a role in HED-guanine excision.

10.1128/mbio.03297-21.3 FIG S3 HedH4 biochemistry and cellular resistance. (A) Coomassie-stained SDS-PAGE of purified HedH4, *S. sahachiroi* AlkZ, and E. coli YcaQ proteins. MW, molecular weight standards. Calculated protein molecular weights are 40.8 kDa (HedH4), 41.2 kDa (AlkZ), and 47.7 kDa (YcaQ). (B) Thermal and enzyme-catalyzed depurination of HED-DNA adducts. Denaturing PAGE of 5′-Cy5-labeled HED-DNA oligodeoxynucleotide substrate and β- and δ-elimination products formed from hydroxide treatment of the abasic site generated from hydrolysis of the HED-deoxyguanosine N-glycosidic bond. Formation of HED-DNA goes to ∼50% completion under our reaction conditions. Lane 1, HED-DNA; lanes 2 and 3, HED-DNA heated to 95°C for 5 min followed by treatment with either water or NaOH; lanes 4 and 5, HED-DNA treated with either buffer (mock) or 1 μM HedH4 for 1 h at 25°C, followed by NaOH. (C) Denaturing PAGE of hedamycin excision by HedH4 wild-type and catalytic mutants Q41A and Q43A. Mock, reaction with buffer alone. Quantification of this gel and the replicates are in Fig. 4D. (D and E) Verification of HedH4 cloning. (D) Agarose gel (1%) of analytical restriction digest of empty pSF-OXB1 and HedH4/pSF-OXB1 using NcoI-HF and XbaI restriction enzymes. Calculated molecular weights for pSF-OXB1 and HedH4 are 3.9 kb and 1.1 kb, respectively. (E) Agarose gel (1%) of colony PCR of HedH4 transformants in E. coli using the HedH4 NcoI and XbaI primers (Table S4). Wild-type E. coli K-12 served as the negative control, while the protein expression vector HedH4/pBG102 served as a positive control. (F and G) Growth curves for WT E. coli K-12 containing either pSF-OXB1 (F) or HedH4/pSF-OXB1 (G) grown in LB/Kan medium supplemented with increasing concentrations of hedamycin. Values are means ± SD (*n* = 3). Download FIG S3, EPS file, 2.7 MB. Copyright © 2022 Bradley et al. 2022 Bradley et al. https://creativecommons.org/licenses/by/4.0/ This content is distributed under the terms of the Creative Commons Attribution 4.0 International license.

We probed specificity of HedH4 for HED-DNA adducts, first by asking whether the HED-guanosine lesion was a substrate for other bacterial alkylpurine DNA glycosylases with various specificities. E. coli AlkA and YcaQ and Bacillus cereus AlkC and AlkD excise a relatively broad range of alkyl-DNA adducts (31, 50–55). *S. sahachiroi* AlkZ, *S. bottropensis* TxnU2 and TxnU4, and *S. vinaceusdrappus* LldU1 and LldU5, like HedH4, are found in BGCs that produce bulky *N*7-alkyl- and intercalating DNA adducts (Fig. 3A), and each is specific for their cognate toxin (31, 56). Compared to HedH4, which excises 100% of the HED-guanine from DNA, none of the 10 alkylpurine DNA glycosylases tested showed any appreciable activity for HED-DNA after 1 h (Fig. 4E). Thus, the HED-DNA adduct is hydrolyzed only by the glycosylase found in the HED BGC. We next examined the ability of HedH4 to excise *N*7-alkylpurine lesions that act as substrates for other YQL and AZL enzymes. Interestingly, HedH4 showed no significant activity for the simple methyl adduct 7mG, which is removed by most alkylpurine DNA glycosylases, including E. coli YcaQ and *S. sahachiroi* AlkZ (Fig. 4F). HedH4 was also unable to hydrolyze TXNA-guanosine, a substrate for TxnU4 from the TXNA BGC (Fig. 4F) (56). We also tested the ability of HedH4 to unhook ICLs derived from AZB (Fig. 1A) and an 8-atom nitrogen mustard, NM_8_ (Fig. 4G), which are substrates for *S. sahachiroi* AlkZ and E. coli YcaQ, respectively. Compared to AlkZ and YcaQ, HedH4 showed little to no activity for either ICL. Thus, HedH4 is highly specific for DNA adducts derived from its cognate natural product.

We next tested if the *hedH4* gene provides heterologous resistance to HED cytotoxicity in cells. E. coli transformed with either vector containing *hedH4* constitutively expressed at low levels or vector alone were grown in the presence of increasing amounts of HED (Fig. S3D to G). HedH4 provided modest protection against HED, as cells expressing HedH4 grew to a higher density at all HED concentrations (Fig. 4I and Fig. S3F and G) and had a higher 50% inhibitory concentration (IC_50_) than cells treated with vector alone (HedH4, 5.9 μM ± 0.7; vector, 3.9 μM ± 0.4). The sensitivity differences between HedH4 and the vector control were more pronounced from a colony dilution assay performed under log-phase growth conditions (Fig. 4J). Cells expressing empty vector displayed an IC_50_ value of 11.1 ± 1.5 μM, while cells expressing HedH4 displayed a 4-fold reduction in sensitivity to HED (48.1 ± 13.8 μM). These results indicate that HedH4 is a DNA glycosylase specific for HED-DNA adducts and provides resistance to cells exposed to the antibiotic.

### YQL proteins from Actinobacteria hydrolyze simple *N*7-alkylguanosine lesions and interstrand cross-links.

**FIG 5:**
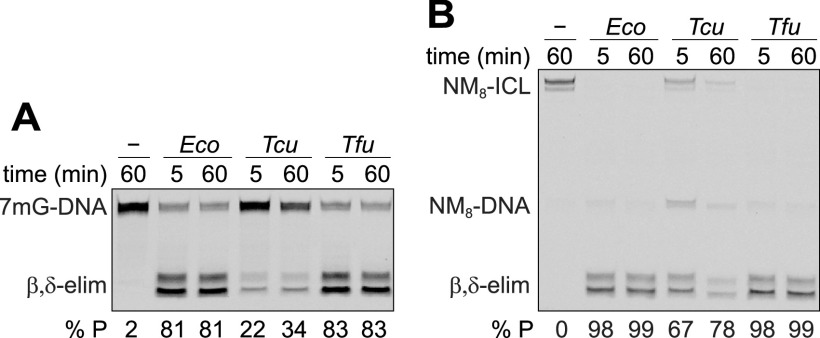
YQL proteins from Actinobacteria hydrolyze simple N7-alkylguanosine lesions and interstrand cross-links. (A and B) Denaturing PAGE of reaction products of E. coli YcaQ (*Eco*) and YQL proteins from Thermomonospora curvata (*Tcu*) and Thermobifida fusca (*Tfu*) with 7mG-DNA (A) and NM_8_-ICL (B) after 5 min and 1 h. Lane 1 of each gel is a no-enzyme control.

We previously characterized E. coli YcaQ to have robust activity toward 7mG and NM-ICLs (Fig. 1B and 4G), a substrate preference distinct from AZB- and HED-specific *S. sahachiroi* AlkZ and HedH4 (Fig. 4F and H) (31). We therefore were interested in determining if other proteins of the YQL subfamily were functional YcaQ orthologs. We purified YQL proteins from the Actinobacteria Thermomonospora curvata and Thermobifida fusca and tested their ability to hydrolyze 7mG and unhook NM_8_-ICLs (Fig. 5). Both proteins showed significant activity for both substrates, providing evidence that the YQL subfamily in general has comparable specificity for simple *N*7-alkylguanine lesions, distinguishing it biochemically from the AZL subfamily.

## DISCUSSION

Phylogenetic characterization of the HTH_42 superfamily proteins within *Streptomyces* reveals two distinct subfamilies, YQL and AZL (the latter of which contains the AZL2 clade). Most strikingly, AZL genes, which are most prevalent in environmental microbes such as those from the phylum Actinobacteria (Fig. S1B), are highly enriched in BGCs. We found AZL proteins in BGCs that produce a variety of verified and putative genotoxins, with approximately one-fifth of all AZL proteins located in BGCs predicted to produce a DNA alkylating agent. We show that the AZL protein, HedH4, within the HED cluster specifically excises HED-DNA adducts and improves viability of cells grown in the presence of the compound. In a separate study, we recently found that the two paralogs present in TXN and LLD clusters (TxnU2, TxnU4, LldU1, and LldU5) are self-resistance glycosylases for these compounds (56). Thus, together with the previous example from the AZB BGC (28, 31), there is now mounting evidence that AZL family genes have evolved largely as DNA repair self-resistance proteins against a variety of natural products. Consistent with their role in resistance, the AZL genes found inside BGCs frequently localize around a variety of other resistance genes. Moreover, the relatively high copy number and low sequence conservation of AZL proteins are consistent with increased expression or possible horizontal gene transfer events that enable these enzymes to evolve specificity for a particular natural product (57). We also found AZL homologs in BGCs that by homology were not expected to produce DNA alkylators or other genotoxins. The AZL proteins in these clusters could have regulatory or protective roles outside DNA repair. Alternatively, these clusters could have additional uncharacterized enzymes such as cytochrome P450s, sulfate adenyltransferases, or epoxidases that could convert the natural products into DNA alkylators (58).

The fate of the AP sites generated by AZL enzymes is a key unanswered question regarding glycosylase-mediated self-resistance in antibiotic bacteria. While the DNA adducts of AZB and HED natural products would likely pose significant blocks to replication and transcription, their excision by AZL glycosylases also generates AP sites, which are highly toxic base excision repair intermediates (59, 60). Although the modest protection we observed from HedH4 overexpression in HED-challenged E. coli could be a result of the weak-expression promoter used, it also suggests that either the AP sites generated are poor substrates for the AP endonucleases present in E. coli or that HED-DNA adducts are substrates for an alternative repair pathway. The intercalated HED-DNA adduct likely poses a unique challenge relative to other glycosylase substrates. It is likely that the HedH4-generated HED-guanine moiety remains intercalated at the AP site and requires a specialized AP endonuclease for repair. Indeed, we recently found that the excised guanine adduct of the related, intercalating natural product TXNA is a poor substrate for E. coli EndoIV (56). More pertinent to HED biosynthesis, the producing organism *S. griseoruber* contains two copies each of ExoIII- and EndoIV-like AP endonucleases that may have evolved to incise HED AP sites, although none are located in the *hed* BGC. In addition, the bulky HED-DNA adduct lesions are likely substrates for the nucleotide excision repair pathway, which is initiated by UvrA in bacteria and has been shown to play an important role in natural product self-resistance (25, 54, 61–63). Indeed, within the HED BGC there is a predicted UvrA-like drug resistance protein (HedH11) that contains a partial UvrA DNA-binding domain and a conserved ABC transporter domain that could initiate NER of HED-guanosine adducts or even HED-guanine/AP-site products generated by HedH4. There are also two additional putative UvrA homologs outside the *hed* cluster. Additionally, there are three putative transporters within the cluster, HedH7 (ABC2 type), HedH6 (DrrA-like), and HedH1 (EmrB/QacA antiporter), which could serve to physically bind to HED and direct it out of the cell through a transmembrane transport system.

In contrast to the genotoxin-specific AZL genes, YQL and AZL2 are always found outside clusters and, thus, are likely to provide a more general role in protecting the genome against environmental genotoxins, similar to that shown for E. coli YcaQ (31). YQL proteins and their gene neighborhoods are very highly conserved, suggesting they play a critical role as part of a unified pathway (64). Although that pathway is unknown, the presence of a two-component transcription factor/kinase and ComF DNA helicase within the YQL neighborhood in *Streptomyces* also hints at a signaling network for DNA uptake (65–67). Similarly, E. coli YcaQ is localized in a four-gene operon involved in cell wall biosynthesis and transformation competence (31). Continued exploration of the gene neighborhoods of YQL and AZL beyond *Streptomyces* will reveal a deeper understanding of the cellular roles played by these enzymes. This will be especially important for YQL, which are prevalent in human pathogens or commensal microbes (28).

A small subset of HTH_42 proteins contain additional domains often associated with nucleic acid transactions (Fig. S1A) (28). These multimodular HTH_42 proteins have been relatively understudied, although they do not appear to be associated with BGCs. Most contain an associated DEAD box helicase domain, including Lhr, a member of the helicase superfamily II (68). Mycobacterium smegmatis and E. coli Lhr have been characterized as ATP-dependent 3′→5′ single-stranded DNA translocases with the ability to unwind RNA-DNA hybrids (69, 70). Studies in Mycobacterium tuberculosis have demonstrated a strong transcriptional activation of *lhr* in cells exposed to MMC (71), suggesting that Lhr functions as an RNA-DNA helicase in response to MMC-DNA cross-links. While the structure of the C-terminal HTH_42 domain of M. smegmatis Lhr is similar to that of AlkZ, it lacks the catalytic QΦQ motif and adopts a tetrameric structure that occludes the putative DNA binding surface (70). Thus, the function of the Lhr HTH_42 domain and its interplay with the helicase core remains to be determined.

Resistance genome mining has emerged as a critical bioinformatically driven pipeline to discover novel natural products and gene clusters in several organisms (72, 73). A key benefit of resistance genome mining is the dramatically decreased candidate pool as a result of targeted identification of gene clusters containing a resistance gene. Generally, these methods require a basic understanding of the resistance mechanisms involved. We sought to use this approach for the first time to hunt for BGCs that produce alkylating genotoxins, using prior knowledge of the DNA repair functions of *S. sahachiroi* AlkZ within the AZB cluster (28, 30, 31). In this study, we examined 435 *Streptomyces* species for BGCs within which an AlkZ-related gene was located and found 68 uncharacterized clusters that are candidates for targeted elucidation of their products. Characterization of these orphan clusters could provide new analogs or types of DNA alkylating/damaging secondary metabolites, an important step in developing new antitumor or antibiotic treatments. This classification of YQL/AZL proteins in *Streptomyces* is an important first step in understanding their evolutionary connection to each other and to BGCs of different types and demonstrates that targeted resistance genome mining is a viable approach to discover novel genotoxins and resistance mechanisms from uncharacterized BGCs.

## MATERIALS AND METHODS

### Reagents.

DNA oligonucleotides (see Table S4 in the supplemental material) were purchased from Integrated DNA Technologies. Escherichia coli K-12 wild-type strain was purchased from the Keio E. coli knockout collection (Dharmacon, GE Healthcare). HED (Streptomyces griseoruber ATCC 23919) was obtained from the National Cancer Institute’s Developmental Therapeutic Program (NCI DTP) Open Compound Repository (NSC 70929). Trioxacarcin A (TXNA) was isolated from Streptomyces bottropensis NRRL 12051 as described previously (56). AZB was prepared from organic extract of *Streptomyces sahachiroi* (ATCC 33158) as in reference 31. NM_8_ compound was synthesized and purified by the Vanderbilt Molecular Design and Synthesis Center (31). AlkA, AlkC, AlkD, AlkZ, LldU1/5, TxnU2/4, and YcaQ were purified as described previously (30, 31, 52, 56, 74, 75). Unless otherwise noted, all chemicals were purchased from Sigma-Aldrich and all enzymes were purchased from New England Biolabs (NEB).

10.1128/mbio.03297-21.7 TABLE S4 Cellular strains, plasmids, and oligodeoxynucleotides used in this study. All oligonucleotides were dissolved in TE buffer (10 mM Tris·HCl pH 8.0, 1 mM EDTA, pH 8.0) to 200 μM, and the DNA was stored at −20°C (stored in the dark for the Cy5/FAM oligonucleotides). The underlined nucleotide in the 7mG_Top, HED_Top, TXNA_Top, AZB_Top/_Bottom, and NM_8__Top/_Bottom oligonucleotides is the site of the *N*7-alkylguanine lesion. PCR was performed with a primer concentration of 500 nM. Download Table S4, XLSX file, 0.01 MB. Copyright © 2022 Bradley et al. 2022 Bradley et al. https://creativecommons.org/licenses/by/4.0/ This content is distributed under the terms of the Creative Commons Attribution 4.0 International license.

### Taxonomy and phylogeny of *Streptomyces* HTH_42 proteins.

To identify HTH_42 proteins in *Streptomyces*, the protein sequences for YcaQ (GenBank accession number QHB65847.1) and AlkZ (GenBank accession number ABY83174.1) were used for tBLASTn and BLASTp searches (BLAST+ v2.11.0) against all *Streptomyces* genomes (taxid:1883). Searches were run with the BLOSUM62 matrix, 1,000 maximum target sequences, and 0.05 threshold using an e-value and identity cutoff of 10^−4^ and 25%, respectively. All hits were verified for the presence of the (H/Q)Φ(D/Q) catalytic motif, during which the (H/Q)Φ(S/T)(D/E) (AZL2) variant was identified. Truncated genes, poor-sequence-quality genes, and pseudogenes were eliminated. Additional sequences were obtained by searching the Pfam database v33.1 (76) for *Streptomyces* HTH_42 superfamily members (PF06224). Sequences from Pfam were sorted according to their domain classes (see Fig. S1A in the supplemental material), and only sequences from class 1 with >75% coverage were included. Protein sequences were aligned using EMBL-EBI Clustal OmegaW or MAFFT v7 using default parameters (77, 78). The evolutionary history of YQL/AZL sequences was reconstructed using IQTREE2 with default settings (79), and the phylogenetic tree was assembled with the Interactive Tree of Life (v5) phylogeny display tool (80). Sequence logos were generated with WebLogo v2.8.2 (81). The copy number frequency and coincidence of YQL/AZL in the same genome was determined by manually counting the number and identity of homologs in each species. A list of all YQL/AZL/AZL2 proteins and *Streptomyces* genomes analyzed in this study can be found in Table S1.

### Identification of AZL proteins in known biosynthetic gene clusters.

To find AZL proteins in verified and/or published BGCs, we searched MIBiG v2.0 for the AZB BGC (BGC0000960) from *S. sahachiroi* (27, 82), followed by an iterative search using the *MIBiG Hits* function until no more hits were obtained. The homologs TxnU2 and TxnU4 were identified from the initial BLAST search within the deposited NCBI trioxacarcin BGC sequence (83). The homolog within the aclacinomycin BGC was also identified in the initial BLAST search as appearing in proximity to aclacinomycin biosynthesis genes. Closer inspection of the published sequence for the aclacinomycin BGC (GenBank accession number AB008466.1) revealed an AZL protein (Orf1) located immediately 3′ of the cluster (84). A detailed list of the AZL proteins in known BGCs can be found in Table S3.

### Identification of AZL proteins in uncharacterized biosynthetic gene clusters.

To determine the physical distance in base pairs between the genomic coordinates of AZL proteins and those of BGCs present in the genome assemblies of *Streptomyces* (average number of scaffolds, 96.30; minimum, 1; maximum, 1,956), we first predicted the BGCs in each genome using antiSMASH v5.1.0 (38) with the *taxon* parameter set to *bacteria*. Using the BGC sequences identified from antiSMASH and AZL sequences, a custom python script using Biopython (85) determined the shortest base pair distance between the physical location of the YQL/AZL gene and the location of the nearest BGC on the same scaffold (less than 2 Mbp away). To be considered within a BGC, the homolog had to be observed within 5 genes or 2 kb of the nearest cluster. Known Cluster BLAST was performed within antiSMASH to determine the BGC most similar to the unknown clusters, and the result with the highest percentage of similar genes was recorded as the most similar cluster. A detailed list of the genome information, cluster identifiers (IDs), and closest 3′ and/or 5′ BGC can be found in Table S2.

### Gene ontology analysis.

To identify GO terms for nearest neighbors identified through BLAST, Pfam, and MIBiG searches, we randomly chose 40 homologs each of AZL inside BGCs, AZL outside BGCs, and YQL, which represent ∼10% of the sequences for each. Amino acid sequences for the five genes on both sides of the YQL/AZL genes were downloaded from the NCBI database, for a total of 400 neighbors for each of the three classes. Cellular functions of any already annotated genes in the NCBI database were identified and recorded. The downloaded sequences were then run through the GhostKOALA (v2.2) and eggNOG (v5.0) GO annotation databases (86, 87). After known GO terms for all gene neighbors were identified, proteins were categorized by biological processes and molecular functions, and the values for these terms were used to create the GO term distributions. Proteins that had multiple GO terms associated with them were counted into each class of terms. A list of all proteins and their annotated GO terms can be found in Tables S5 and S6.

10.1128/mbio.03297-21.8 TABLE S5 AZL nearest neighbor GO term analysis. Nearest 5 open reading frames (ORFs) upstream (−) (3′→5′) and downstream (+) (5′→3′) of AlkZ-like proteins predicted to be within (A) or outside (B) BGCs. ORFs are listed with their GenBank/RefSeq ID and biological pathway and molecular GO terms, as determined by NCBI, GhostKOALA, and eggNOG databases. Empty cells mean no GO terms could be assigned to these proteins through homology search. Download Table S5, XLSX file, 0.04 MB. Copyright © 2022 Bradley et al. 2022 Bradley et al. https://creativecommons.org/licenses/by/4.0/ This content is distributed under the terms of the Creative Commons Attribution 4.0 International license.

10.1128/mbio.03297-21.9 TABLE S6 YQL nearest neighbor GO term analysis. Nearest 5 open reading frames (ORFs) upstream (−) (3′→5′) and downstream (+) (5′→3′) of YcaQ-like proteins assigned to be outside BGCs. ORFs are listed with their GenBank/RefSeq ID and biological pathway and molecular GO terms as determined by NCBI, GhostKOALA, and eggNOG databases. Empty cells mean no GO terms could be assigned to these proteins through homology search. Download Table S6, XLSX file, 0.02 MB. Copyright © 2022 Bradley et al. 2022 Bradley et al. https://creativecommons.org/licenses/by/4.0/ This content is distributed under the terms of the Creative Commons Attribution 4.0 International license.

### Protein purification.

Genes encoding Streptomyces caeruleatus AZL2, Streptomyces griseoruber HedH4, Thermomonospora curvata YQL, and Thermobifida fusca YQL were codon optimized and synthesized by GenScript and cloned into pBG102. The N-terminal His_6_-SUMO fusion proteins were overexpressed in Escherichia coli Tuner(DE3) cells at 16°C for 18 h in LB medium supplemented with 30 μg/mL kanamycin and 50 μM isopropyl β-d-1-thiogalactopyranoside (IPTG). Cells were lysed by sonication and cell debris removed by centrifugation at 45,000 × *g* at 4°C for 30 min. Clarified lysate was passed over nickel-nitrilotriacetic acid (Ni-NTA) agarose equilibrated in buffer A (50 mM Tris·HCl, pH 8.5, 500 mM NaCl, 25 mM imidazole, and 10% [vol/vol] glycerol) and protein eluted in 250 mM imidazole-buffer A. Protein fractions were pooled and supplemented with 0.1 mM EDTA and 1 mM tris(2-carboxyethyl)phosphine (TCEP) before incubation with 0.5 mg rhinovirus 3C protease (PreScission) at 4°C overnight. Cleaved protein was diluted 10-fold in buffer B (50 mM Tris·HCl, pH 8.5, 10% [vol/vol] glycerol, 0.1 mM TCEP, and 0.1 mM EDTA) and purified by heparin Sepharose using a 0 to 1 M NaCl-buffer B linear gradient. Fractions were pooled and passed over Ni-NTA agarose in buffer A, concentrated and filtered, and buffer exchanged into buffer C (20 mM Tris·HCl, pH 8.5, 100 mM NaCl, 5% [vol/vol] glycerol, 0.1 mM TCEP, and 0.1 mM EDTA). Protein was concentrated to 4 mg/mL, flash-frozen in liquid nitrogen, and stored at −80°C. Mutant protein expression vectors were generated using the Q5 mutagenesis kit (New England BioLabs), and proteins were overexpressed and purified the same as the wild type.

### DNA glycosylase activity.

DNA substrates containing a single *N*7-methyl-2′-deoxyguanosine lesion and a 5′-Cy5 fluorophore were prepared as described previously (88). AZB- and NM_8_-ICL substrates were generated and purified as in reference 31. DNA substrates containing a single HED-guanosine or trioxacarcin A (TXNA)-guanosine adduct were prepared by annealing 5′-Cy5-labeled DNA containing the target sequence to the complementary unlabeled oligodeoxynucleotide (Table S4). HED and TXNA were dissolved in dimethyl sulfoxide (DMSO) to a concentration of 5 mM, and 100 μM DNA was incubated with 200 μM HED or TXNA in 10% methanol and 20% DMSO at 4°C on ice in the dark for 24 h. Unreacted drug was removed using an Illustra G-25 spin column (GE Healthcare) equilibrated in TE buffer (10 mM Tris·HCl, pH 8.0, 1 mM EDTA, pH 8.0), and the DNA was stored at −80°C.

In each glycosylase reaction, 1 μM enzyme was incubated with 50 nM DNA in glycosylase buffer (50 mM HEPES, pH 8.5, 100 mM KCl, 1 mM EDTA, and 10% [vol/vol] glycerol) at 25°C. At various time points, 4-μL aliquots were added to 1 μL of 1 M NaOH and heated at 70°C for 2 min. Samples were denatured at 70°C for 5 min in 5 mM EDTA, pH 8.0, 80% (wt/vol) formamide, and 1 mg/mL blue dextran prior to electrophoresis on a 20% (wt/vol) acrylamide–8 M urea sequencing gel at 40 W for 1 h in 0.5× TBE buffer (45 mM Tris, 45 mM borate, and 1 mM EDTA, pH 8.0). Gels were imaged on a Typhoon Trio variable-mode imager (GE Healthcare) using 633-nm excitation/670-nm emission fluorescence for Cy5, and bands were quantified with ImageQuant (GE Healthcare). All excision assays were performed in triplicate.

### HPLC-MS analysis of HED and HED-guanine.

HPLC was performed on an Agilent Series 1100 system equipped with an analytical SymmetryShield RP-C_18_ column (3.5 μm, 4.6 mm by 7.5 mm, 100-Å pore size) and using a linear gradient from 90% buffer A (10 mM ammonium formate)–10% buffer B (100% methanol) to 100% B over 40 min and a flow rate of 0.4 mL/min. HED was diluted to 50 μM in 10% methanol and stored on ice prior to HPLC injection. To analyze the product of HedH4 activity, HED-DNA was diluted to 10 μM in glycosylase buffer and reacted with 50 μM HedH4 for 1 h at room temperature before injection. Mass spectrometry was performed with an LTQ Orbitrap XL hybrid FT mass spectrometer (Thermo Fisher Scientific) in positive ion mode from 300 to 1,000 *m/z*.

### Cellular assays for HED resistance.

The *hedH4* wild-type gene was subcloned from pBG102 into pSF-OXB1 using NcoI and XbaI restriction sites. The pSF-OXB1 vector contains a kanamycin resistance gene and allows for constitutive low-level expression from a modified AraBAD promoter. pSF-OXB1 and HedH4/pSF-OXB1 were transformed into E. coli K-12 cells. Cloning of *hedH4* was confirmed by sequencing, restriction digest using NcoI-HF/XbaI (Fig. S4C), and colony PCR of K-12 transformants using the HedH4 NcoI and XbaI primers (Fig. S4D, Table S4). Cultures were grown at 37°C in LB medium supplemented with 30 μg/mL Kan. Growth curves were generated by diluting overnight cultures to an optical density at 600 nm (OD_600_) of 0.01 in LB/Kan supplemented with 0 nM to 100 μM HED in a 96-well flat-bottom plate. The plate was incubated at 30°C with shaking for 24 h, and cell density was measured at 600 nm every 20 min using a Bio-Tek Synergy 2 microplate reader. IC_50_ values were determined from a fit to the equation lag time = min_lag_ + (max_lag_ − min_lag_)/[1 + (IC_50_/[HED])*^h^*], where *h* is the Hill slope. Growth experiments were performed in triplicate.

E. coli survival curves after HED treatment were performed using a colony dilution assay. A saturated overnight LB/Kan culture from a single colony was diluted to an OD_600_ of 0.01 in 1 mL fresh LB/Kan medium and grown to an OD_600_ of 0.6 at 37°C. The cells were treated with various concentrations of HED for 1 h at 37°C. Treated cells were transferred to fresh LB/Kan medium and serially diluted by 10^−6^ in LB/Kan medium, and 100 μL of diluted cells was plated on LB/Kan agar plates and grown at 37°C overnight. Colonies were counted the next morning and the number of CFU/mL culture was determined. The percent survival was calculated as CFU/mL (treated) divided by CFU/mL (untreated). Curves were plotted on a logarithmic scale and IC_50_ values determined by nonlinear regression fits to the data. Growth experiments were performed in triplicate.
